# Carcinoembryonic Antigen-Related Cell Adhesion Molecules (CEACAM) 1, 5 and 6 as Biomarkers in Pancreatic Cancer

**DOI:** 10.1371/journal.pone.0113023

**Published:** 2014-11-19

**Authors:** Florian Gebauer, Daniel Wicklein, Jennifer Horst, Philipp Sundermann, Hanna Maar, Thomas Streichert, Michael Tachezy, Jakob R. Izbicki, Maximilian Bockhorn, Udo Schumacher

**Affiliations:** 1 Department of General, Visceral and Thoracic Surgery, University Medical Center Hamburg-Eppendorf, University of Hamburg, Hamburg, Germany; 2 Institute of Anatomy and Experimental Morphology and University Cancer Center Hamburg (UCCH), University Medical-Center Hamburg-Eppendorf, Hamburg, Germany; 3 Institute of Clinical Chemistry, University Medical-Center Hamburg-Eppendorf, Hamburg, Germany; Wayne State University School of Medicine, United States of America

## Abstract

**Background:**

Aim of this study was to assess the biological function in tumor progression and metastatic process carcinoembryonic antigen-related cell adhesion molecules (CEACAM) 1, 5 and 6 in pancreatic adenocarcinoma (PDAC).

**Experimental Design:**

CEACAM knock down cells were established and assessed in vitro and in a subcutaneous and intraperitoneal mouse xenograft model. Tissue and serum expression of patients with PDAC were assessed by immunohistochemistry (IHC) and by enzyme linked immunosorbent assays.

**Results:**

Presence of lymph node metastasis was correlated with CEACAM 5 and 6 expression (determined by IHC) and tumor recurrence exclusively with CEACAM 6. Patients with CEACAM 5 and 6 expression showed a significantly shortened OS in Kaplan-Meier survival analyses. Elevated CEACAM6 serum values showed a correlation with distant metastasis and. Survival analysis revealed a prolonged OS for patients with low serum CEACAM 1 values. In vitro proliferation and migration capacity was increased in CEACAM knock down PDAC cells, however, mice inoculated with CEACAM knock down cells showed a prolonged overall-survival (OS). The number of spontaneous pulmonary metastasis was increased in the CEACAM knock down group.

**Conclusion:**

The effects mediated by CEACAM expression in PDAC are complex, though overexpression is correlated with loco-regional aggressive tumor growth. However, loss of CEACAM can be considered as a part of epithelial-mesenchymal transition and is therefore of rather importance in the process of distant metastasis.

## Introduction

In recent years, the prognosis of patients suffering from pancreatic ductal adenocarcinoma (PDAC) has not improved significantly [1], [2]. Most patients present with advanced tumor stages making curative treatment impossible. Complete surgical resection, followed by adjuvant chemotherapy is nowadays the gold-standard in therapy of PDAC. However, even in patients after complete surgical tumor resection, the overall survival remains poor with a median survival time of 20–24 months [3]–[5]. Aggressive loco-regional tumor growth in addition to early distant and peritoneal metastasis and a high degree of chemoresistance make PDAC one of the most lethal gastrointestinal tumors [6].

Today, neither reliable serum nor tissue markers predicting the clinical course of patients after diagnosis of PDAC are available. Furthermore, the molecular interactions of the tumor with the host and the local factors that allow PDAC to display such an aggressive progression are poorly understood. Therefore, there is an imperative need for a better understanding of the tumor biology with respect to the mechanisms of local tumor invasion and recurrence.

Carcioembryonic antigen-related cell adhesion molecules (CEACAMs) are members of the glycosylphosphatidylinositol (GPI)-linked immunoglobulin (Ig) superfamily [7]. There are more than 17 genes that belong to this family, with their gene products primarily integrated into the cell membrane. Within the CEACAM family the CEACAM subtypes are structurally similar and physiologically expressed on the apical surface of numerous cell types, e.g. endothelial and hematopoietic cells as well as epithelial cells of different organs. Depending on the cell type and CEACAM subtype, the transmitted effect after binding a certain partner varies, including regulation of cell adhesion, tumor suppression, angiogenesis, activation of leukocytes and other immuno-reactive cells, and regulation of the cell cycle [8]–[11]. The CEACAM 5 gene, its product also known as CD66e codes for the carcioembryonic antigen (CEA) and has become one of the best-known members of the Ig-superfamily since it has a significant role in the clinical routine as a tumor marker for several tumor entities including gastrointestinal and respiratory malignancies [12]. However, due to lacking sensitivity and specificity its predictive value alone is still unsatisfying [13]–[16]. The CEACAM subtypes 1 and 6 are described to be under- or overexpressed in several tumor entities like lung cancer, colon cancer and melanoma [17]–[23]. Though overexpression is widely observed, some studies even report a decreased expression in certain tumor entities at different tumor stages.

Interestingly, recent studies found CEACAM 1 and 6 expression in primary PDAC correlated with a shortened overall patient survival [24], [25]. The biological principles why CEACAM expression mediates tumor progression are not fully understood. We therefore analyzed the effects of CEACAM 1, 5 and 6 in vitro and established a xenograft mouse model to investigate the functional role of CEACAM expression in PDAC. To assess whether CEACAM expression has an impact on the tumor progression in patients, we combined serum and immunohistochemical analysis for CEACAM molecules 1, 5 and 6 to analyze whether there is a correlation of CECACM expression with clinico-pathologcial data of patients with PDAC.

## Material and Methods

### Cell line and CEACAM knock down

The human pancreatic adenocarcinoma cell line PaCa 5061 was established from a primary tumor. Detailed characteristics of establishment and culture of the cell line have been described previously [26]. Briefly, cells were obtained from a patient with a PDAC who underwent surgical resection in the Department of General, Visceral and Thoracic Surgery at the University Medical Center Hamburg-Eppendorf. The final tumor classification according to the UICC 7^th^ ed. revealed a pT3, N1, L1, V1, R0, G2 PDAC of the pancreatic head.

CEACAM knock down variants were conducted by shRNA interference as described previously [27]. Oligonucleotides were cloned into a pSIREN-RetroQ vector and transfected by FuGENE transfection agents (Roche Diagnostics, Hilden, Germany) with the tumor cells. Selection of knock down clones was performed by expression of a Puromycin chemoresistance (Clontech, Saint-Germain-en-Laye, France) and flow-cytometer sorting (FACS-LSR Fortessa, BD Bioscience, San Jose, USA). Only cells with a knock down of >90%, as determined by flow cytometry, were used for in vitro and in vivo experiments. Unconjugated antibodies against CEACAM 1, 5 and 6 and the corresponding mouse biotinylated IgM or rat IgM isotype control (Dako, Glostrup, Denmark) were detected with goat anti-mouse Ig-APC (BD Biosciences). Cells were analyzed using a CyFlow cytometer (Partek, Münster, Germany) with a subsequent addition of AlexaFluor488 conjugated streptavidin (Invitrogen) before staining.

### In vitro characterization of CEACAM knock down cells

Cell proliferation was assessed using colorimetric XTT assay (Roche Diagnostics, Basel, Switzerland) according to manufacturer's instructions. Cells were plated in 96-well plates at 3000 cells/well. After 48 h to allow for cell adherence, cells were incubated with colorimetric substrates. Colorimetric changes were measured in a multi-well spectrophotometer (MR5000 Multiplate Reader, Dynatech, Denkendorf, Germany).

Differences in cell migration were assessed using FluoroBlok Migration Assay (BD Bioscience, San Jose, USA) with 24-well 8 micron pore size inserts. Cells were trypsinized and re-suspended in serum-free RPMI1640 (Invitrogen, Darmstadt, Germany) in a concentration of 300.000 cells/mL. 400 µl cell suspension was added to the apical chamber and 800 µl RPMI1640 with 10% fetal calf serum (FCS, Invitrogen) was added to the bottom chamber. The assay was incubated for 24 h under standard cell culture conditions.

After removal of the chemo attractant of the bottom chamber, visualization of migrated cells was performed by adding 500 µl/well HBSS buffer with Calcein AM (Invitrogen) 4 µg/mL in the bottom well and incubation for 1 h. Readout was conducted at 494/517 nm (Ex/Em) on a Genios bottom-reading fluorescence plate reader (Tecan, Männedorf, Swizerland).

Laminar flow experiments were performed using IBIDI microslides VI (IBIDI, Munich, Germany) connected to a syringe pump (Model 100 Series; kdScientific, Holliston, MA) and cell movement was observed with an inverted microscope (Zeiss, Jena, Germany; Axiovert 200). Tumor cells were suspended in cell culture medium (20 ml, 200 000 cells/ml) and microslides were coated with Human Pulmonary Microvascular Endothelial Cells (HPMEC) (PromoCell, Heidelberg, Germany). HPMEC were suspended in cell culture medium, seeded in microslides at a concentration of 5×10^5^ cells/ml and 20 µl medium with cells was pipetted into each flow channel. Cell grew confluent over night under standard conditions. Applied shear rates ranged from 0.05 dyn/cm^2^ to 10.0 dyn/cm^2^. Cell movement was recorded and analyzed with regard to the quality of movement (adhesion, rolling and tethering) and rolling velocity using CapImage 8.5 program (Dr. Heinrich Zeintl, Heidelberg, Germany).

### Animal experiments

Animal experiments were conducted according to the UKCCR guidelines for the welfare of animals in experimental neoplasia [28], the locals Ethics committee for animal experiments (Behörde für Soziales, Familie, Gesundheit, Verbraucherschutz; Amt für Gesundheit und Verbraucherschutz; Billstr. 80, D-20539 Hamburg, Germany, project No. G58/09) and as well the institutional animal welfare officer of the University Medical-Center recommended and approved the study.

For the subcutaneous tumor model, one million PaCa 5061 tumor cells were injected in the right scapula region in 8–12 week old C57BL/6N pfp^-/-^/rag2^-/-^ double-knockout mice. Animals were sacrificed when primary tumors exceeded 2 cm^3^ or ulcerated the mouse skin, the mice were terminally narcotized and sacrificed by cardiocentesis.

For assessment of the influence of CEACAM expression on peritoneal dissemination, we established an intraperiteoneal tumor model as previously described [29]. Briefly, one million tumor cells were injected in the lower left abdominal quadrant intraperitoneally in suspension volume of 200 µL. Assessment and time points of termination of the experiment was conducted according to a previous established scoring system. Assessment of the extend of the intraperitoneal tumor growth was done with a modified peritoneal carcinomatosis index (PCI) as described previously [30]. Briefly, the peritoneal cavity is divided in 9 abdomino-pelvic regions, depending on the extend of tumor growth, scores between 0 and 3 points are assigned (0 points: no tumor present; 1 point: tumor <1 mm^3^; 2 points: tumor >1 and <3 mm^3^; 3 points: tumor >3 mm^3^) that lead to a PCI score from 0 to 27.

### Quantification of pulmonary metastasis, disseminated tumor cells (DTC) and CTC by Alu-PCR

The left lungs were homogenized in a sample disruptor (TissueLyser II, Qiagen, Hilden, Germany) and subjected to DNA-isolation (QIAamp DNA Mini Kit, Qiagen). Bone marrow was collected by flushing the left femora with 1 ml NaCl 0.9%. 200 µl blood and the bone marrow suspensions were subjected to DNA- isolation using the QIAamp DNA Blood Mini Kit.

DNA concentrations of all samples were quantified using a NanoDrop spectrophotometer (Peqlab, Erlangen, Germany). As the content of detectable Alu-sequences in the following qPCR would have been affected simply by varying DNA-concentrations, all lung- and bone marrow-DNA samples were normalized to 30 ng/µl using AE buffer (Qiagen). The concentrations of blood-DNA were quite similar in all samples (approx. 10 ng/µl) and were therefore not normalized. qPCR was performed with established human-specific Alu-primers [31]. 2 µl total DNA (i.e., 60 ng lung/bone marrow-DNA; 20 ng blood-DNA) were used for each qPCR. Numerical data were determined against a standard curve as described [32]. The detection limit for specific human Alu-sequence signals was determined for each tissue type by testing DNA from five healthy (non-injected) pfp^-/-^/rag2^-/-^ mice of similar sex and age. For each sample, analyses were performed in duplicates and as independent experiments at least twice.

### Patients and surgical procedures

Between 1992 and 2009, all patients who underwent major resectional pancreatic surgery at the Department of General, Visceral and Thoracic Surgery at the University Medical Centre Hamburg-Eppendorf were included in a prospective, pancreatic database. The study was approved by the Ethics Committee of the Chamber of Physicians in Hamburg, Germany. Written consent for using the samples for research purposes was obtained from all patients prior to surgery or blood drawing.

Patients with PDAC of the pancreatic head region routinely underwent either partial pancreatoduodenectomy (PD) or pylorus-preserving duodenopancreatectomy (PPDP) and organ-preserving resection methods in cases of chronic pancreatitis (CP). Only patients with macroscopic complete tumor resection were included in the final analysis. In-hospital mortality was defined as death at any time during the entire period of hospitalization. Follow-up information was obtained from our institution's outpatient clinic, from the appropriate general practitioners' offices, or from the regional cancer registry. When the date of death was not recorded, patients were censored at the last recorded contact.

### Tissue micro array construction and immounohistochemistry

Tissue cores were obtained from formalin-fixed paraffin-embedded (FFPE) tissue blocks from patients with pathologically proven PDAC. Representative areas of the tumor were selected based on hematoxylin-eosin staining.

TMA construction was performed as previously described [33]–[35]. Briefly, 252 tissue cylinders with a diameter of 0.6 mm were punched from the “donor” tissue blocks using a custom-made semiautomatic robotic precision instrument and placed into one paraffin block that contained the 252 individual samples. Within these samples, there were 142 PDACs, 40 neuroendocrine pancreatic tumors (NET), 33 intraductal papillary mucinous neoplasm (IPMN), and 37 samples of healthy tissue as a negative control. The resulting TMA blocks were used to produce 4-µm sections that were transferred to an adhesive-coated slide system (Instrumedics Inc., Hackensack, New Jersey, USA).

The immunohistochemical staining protocols were optimized on various benign and malignant tissues in an extensive multistep procedure that modified the staining protocol until the required selective staining was achieved with the lowest possible background signal (according to [36]).

Sections were deparaffinized and dried overnight at 37°C. Antigen retrieval was performed by microwave oven treatment in citrate buffer (pH 6.0) for 1 min, sections were then rehydrated in Tris-buffered saline (TBS; 0.05 M Tris-HCl at pH 7.6 and 0.15 M NaCl) and blocked with rabbit AB serum (Biotest Diagnostics, Dreirach, Germany) diluted 1∶10 in TBS for 60 minutes. CEACAM staining was performed using a specific CEACAM monoclonal antibody (CEACAM1 clone 4D1/C2 IgG2a, in-house clone (previously described by [37]) at a dilution of 1∶200, CEACAM5 clone #2383 IgG1 at a dilution of 1∶50 (Cell Signaling, Beverly, USA); CEACAM6 clone IgG1 (9A6), at a dilution of 1∶40 [Sigma Aldrich, Hamburg, Germany]) overnight at 4°C. Biotinylated secondary polyclonal rabbit anti-mouse antibodies (Dako, Hamburg, Germany) were used for binding the CEACAM primary antibody. Epithelial-mesenchymal transistion (EMT) markers were studies by immunohistochemistry as well. ZEB 1 (IgG at a dilution of 1∶100, polyclonal rabbit anti-human, Atlas Antibodies, Stockholm, Sweden), ZEB 2 (IgG at a dilution of 1∶100, polyclonal rabbit anti-human, Atlas Antibodies, Stockholm, Sweden), E-Cadherin (), Pan-Cytokeratin ().

The binding sites were detected using the ABC-AP-Kit (Vector Laboratories Inc., Burlingame, USA). Alkaline phosphatase activity of a biotin-streptavidin–alkaline phosphatase complex was visualized using naphthol-AS bisphosphate as substrate and hexatozised New Fuchsin was used for simultaneous coupling. The sections were counterstained with Mayer's hemalum (Merck, Darmstadt, Germany).

The staining intensity (0, 1+, 2+, 3+) and the fraction of positive tumor cells were scored for each tissue spot as recently published [34]. Spots without staining and with a staining intensity of 1+ in <70% and 2+ in <30% of the tumor cells were scored as CEACAM low, medium scores were given for a staining intensity of 1+ in ≥70%, 2+ in ≥30% or 3+ in <30% of the tumor cells, and high scores were given for a staining intensity of 2+ in ≥70% or 3+ in ≥30% of the tumor cells. Immunohistochemical analysis of the sections was performed without knowledge of the patients' identity or clinical status. Immunohistochemical analysis and scoring were performed by two independent investigators who were unaware of the patient outcome or other clinical findings. In 95% of the samples, the evaluations of the two observers were identical, the remaining slides were re-evaluated, and consensus decisions were made.

The staining protocol was as well used for mice grown tumors and showed similar sensitivity and specificity compared to the TMA staining.

### Enzyme linked immunosorbent assay (ELISA)

For quantification CEACAM subtypes in peripheral blood, serum samples of 46 Caucasian patients with PDAC and 47 Caucasian patients with CP, who were indicated for surgical treatment, were analyzed with an enzyme linked immunoassay (ELISA). All blood samples were obtained directly before surgery. As healthy controls, 50 Caucasian blood-bank donors, obtained from the institute for transfusion medicine (University Medical Centre Hamburg-Eppendorf), were included in the study. Preparation of serum samples were conducted according to a standardized protocol [38]. Median age was 62.4 years at time of diagnosis (range 36.3–90.4 years, 24 male [52.2%], 21 female [47.8%]). Serum values of 47 patients who underwent surgery due to chronic pancreatitis (median age at time of diagnosis 47.0 years, range 31.1–76.1 years) and 50 samples of healthy blood donors were used as controls (25 male [50%], 25 female [50%]).

For the detection of CEACAM 1 and CEACAM 5 in the serum, 96-well flexible microtiter plates (Costar 9019, USA) were coated with 50 µl per well of 2 µg/ml of monoclonal mouse capture antibody (Clone 283324 and 843130, mouse IgG1, R&D systems, USA) overnight at 4°C. Wells were blocked with 3% w/v bovine serum albumin (BSA; Fraction V, 98% purity, Sigma Aldrich, Germany) in PBS/T (phosphate buffered saline, pH 7.3, containing 0.05% v/v Tween) for 45 min at room temperature and then incubated for 1 h with human sera diluted 1∶50 in PBS at room temperature. After five washes with PBS/T, bound protein was detected with biotin-conjugated polyclonal antibody anti-CEACAM 1 or 5 (CEACAM 1 Goat IgG, clone 842284; CEACAM 5 sheep IgG, clone 843131Goat IgG, R&D systems, USA), respectively, followed by streptavidin-conjugated peroxidase using TMB (3,3′, 5,5″-tetramethylbenzidine) as substrate. The color reaction was stopped by addition of 10 mM H_2_SO_4_ and analyzed at 450 nm using an ELISA reader (DynaTech MR 5000, USA). Human recombinant CEACAM 1 and 5 proteins (R&D systems) served as an internal standard for the assay.

CEACAM 6 was determined by a direct ELISA coating 50 µl patient serum in a 1∶50 dilution over night a 4°C in the 96-well plate. Wells were blocked with 3% bovine serum albumin (BSA; Fraction V, 98% purity, Sigma Aldrich) in PBS/T (phosphate buffered saline, pH 7.3, containing 0.05% v/v Tween) for 45 min at room temperature and then incubated for 1 h with the detection antibody anti-CEACAM 6 (mouse IgG1, clone 843158, R&D systems). The color reaction was stopped by addition of 10 mM H_2_SO_4_ and analyzed at 450 nm. To ensure that the immunoassay was suitable for measuring clinical serum samples, reproducibility and linearity were examined (according to [39]).

### Statistical analysis

For explorative statistical analysis of the individual patient groups, either a two-sided chi-square test or a Fisher exact test was used. Quantitative variables were either tested by means of the Student *t-test* or by medians of the Wilcoxon test. Test for normal distribution of the quantitative variables was performed by Kolmogorov-Smirnov-Test. Kaplan–Meier analysis (log-rank test) was used for disease free- and overall-survival analysis excluding in-hospital mortality. All variables achieving a P value ≤0.05 were included in a multivariate cox-regression model. The cut-off level of serum CEACAM quantification was determined by using the Youden-index and as described previously [33], [40].

## Results

### In vitro characteristics of CEACAM knock down PDAC cells

**Figure 1:**
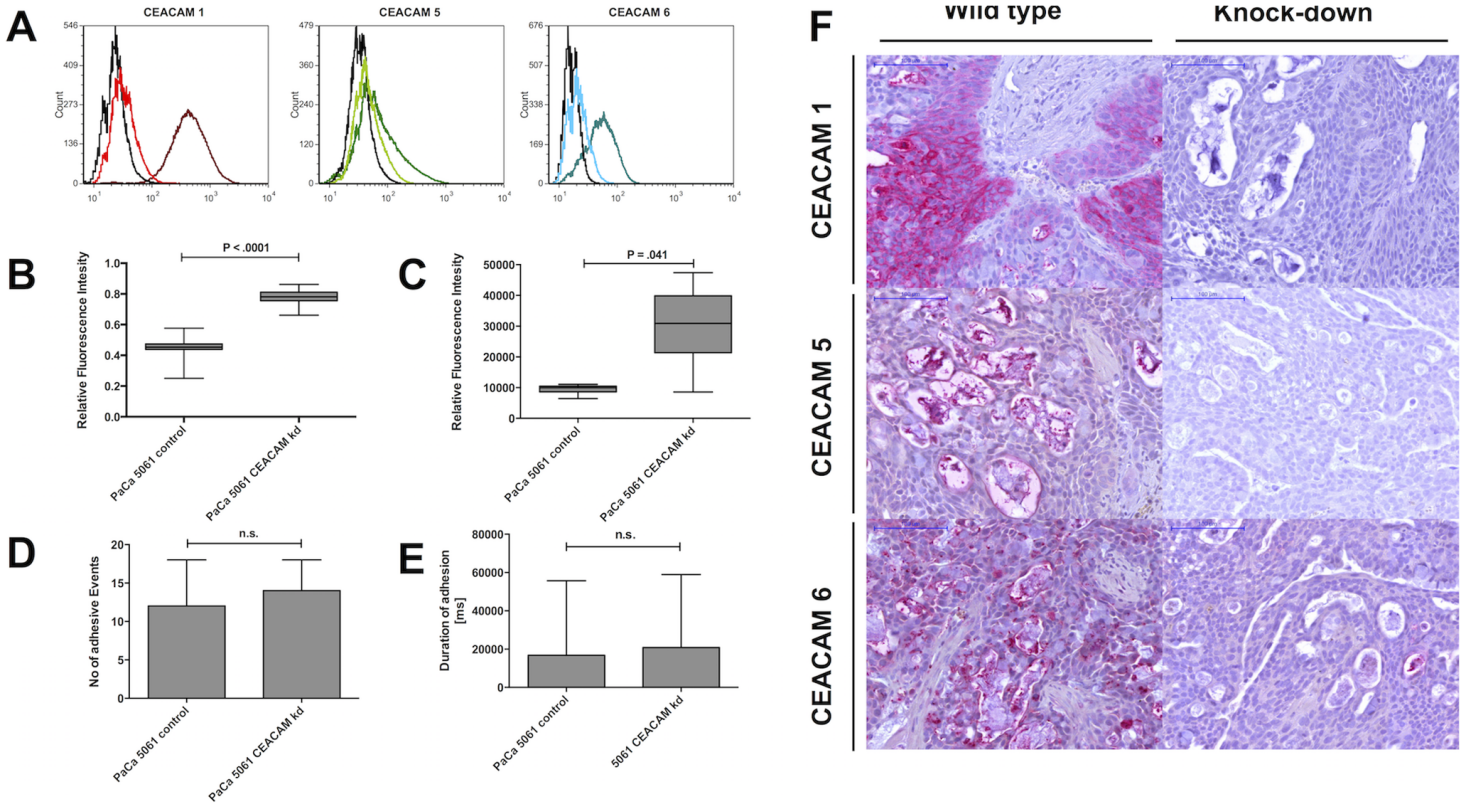
CEACAM levels decreased to <5% for CEACAM 1, 5 and 6 after shRNA knock down as shown in flow cytometry. (A). Basal CEACAM expression varied depending on the CEACAM subtype, highest levels were found for CEACAM 1, intermediate for CEACAM 6 and lowest levels for CEACAM 5. As seen in proliferation (B) and migration assays (C), CEACAM knock down cells showed a higher proliferative and migrative potential compared to control cells. Differences in adhesion on stimulated (TNFα) endothelial cells were not detectable between CEACAM kd and control cells. Neither the average number (D) nor the average adhesion time to the endothelial cells was different (E). Murine xenograft tumors were highly positive for CEACAM 1, 5 and 6. Immunohistochemical staining in control cells showed complete absence of CEACAM expression in immunohistochemistry in knock down cells (F).

As analyzed by flow cytometry, basal CEACAM expression was present on the tumor cells. Surface levels of the CEACAM subtypes 1, 5 and 6 were reduced to <5% compared with CEACAM expression on control cells (Figure 1A). Proliferation and migration increased in the CEACAM knock down cells compared with the control cells (Figure 1B and C), but CEACAM knockdown did not affect the adherence on stimulated endothelium (HPMEC) (Figure 1D and E).

For control of presence of the CEACAM knock down in the murine grown tumors, IHC staining of CEACAM 1, 5 and 6 was performed and, as depicted in Figure 1F, the knock down levels were stable and still present in the tumors grown in the mice.

### Xenograft model for functional analysis of CEACAMs in PDAC

**Figure 2:**
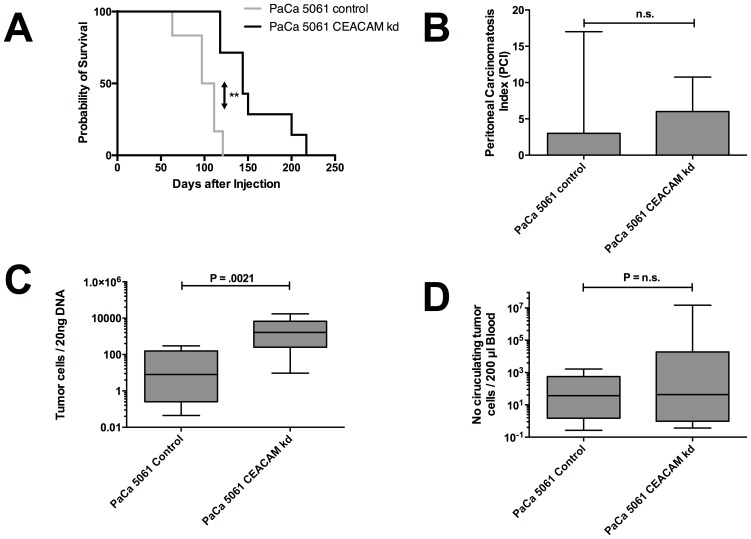
Subcutaneous murine xenograft model showed prolonged survival in CEACAM knock down group compared to the control group (A). Intraperitoneally, no differences in the peritoneal carcinomatosis index (PCI) were observed at time of scarification (80 days after injection). CEACAM knock down showed more DNA copies of human DNA in the left lung (P = 0.021) (C) which is correlated with a higher metastatic load in the lung, however, no difference was observed for circulating tumor cells in the blood (D).

To answer the question whether CEACAM family members have a functional effect on tumor formation, growth and metastasis, a tumor xenograft experiment with human PDAC cells (with and without CEACAM knockdown) in pfp^–^/rag2^–^ mice was performed. After subcutaneous tumor cell injection, mice inoculated with CEACAM knock down cells showed a significantly prolonged overall survival until reaching the termination criteria (median survival 144 d) when compared with PaCa 5061 control (median survival 104 d; P = 0.014) and wild-type PACA 5061 (median survival 79 d; P = 0.01) (Figure 2A). The tumor size and weight at time of death did not differ significantly between the groups (data not shown).

While the subcutaneous xenograft model showed a prolonged OS for mice with CEACAM knockdown, the influence of the CEACAM knock down in the intraperitoneally xenograft model was not present. The peritoneal carcinomatosis index did not differ between the groups, which was also represented in missing differences in the dissemination as there were no differences between the CEACAM knock down and the control group in circulating tumor cells in peripheral blood (Figure 2B and D). However, in the CEACAM knock down group, we found higher amounts of human DNA (meaning significantly more PDAC cells) in the lungs compared with the control group (Figure 2C). PDAC cells showed no affinity for dissemination to the bone marrow, human tumor cell DNA was not detected in any of the groups. Markers for EMT showed no significant differences between the wild type and CEACAM knock down tumors as determined by immunohistochemistry (Fig S1).

### Immunohistochemical CEACAM 1, 5 and 6 expression in patients samples

Out of the 142 tumor spots, 5 (3.5%) specimen were not evaluable on the TMA due to missing or unrepresentative tumor tissue. Tissue specimens of 137 patients were finally evaluable and correlated with clinic-pathological data. Patients were aged between 33.1–85.0 years (median 63.5 years). There were 82 male patients (59.9%) and 55 female (40.1%).

**Figure 3:**
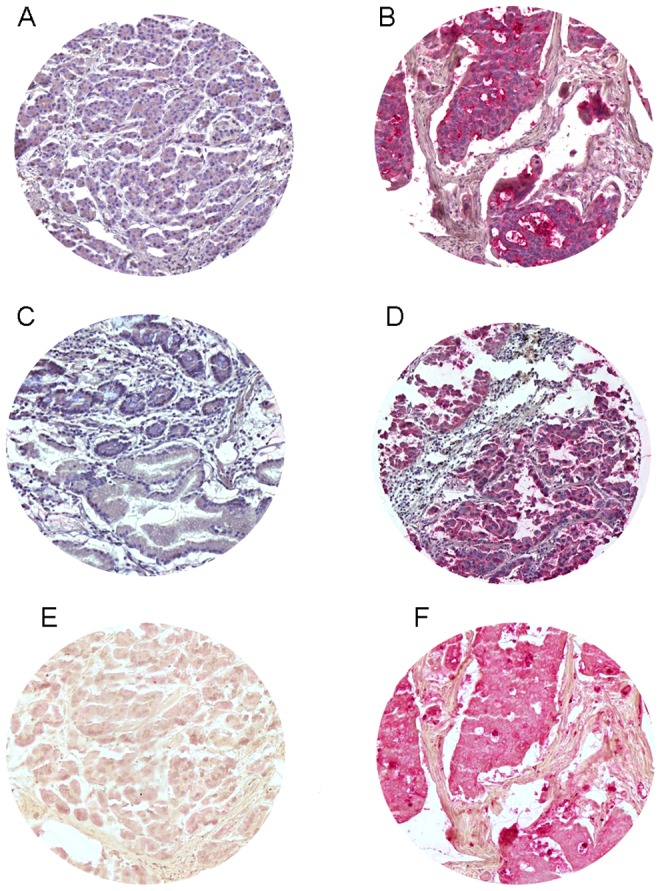
TMA immunohistochemical staining of CEACAM 1 negative (A) and positive (B), CEACAM 5 negative (C) and positive (D), CEACAM 6 negative (E) and positive (F).

CEACAM 1 expression was found in 62.8% of all tumor specimens (n = 86), CEACAM 5 in 87 patients (63.5%), CEACAM 6 expression was observed in 99 patients (72.3%). The majority of the tumors showed a homogeneous staining within each specimen, though in a minority of tumor specimens, we observed an inhomogeneous IHC staining pattern within the tumor area. The expression pattern of all three CEACAMs was membranous and cytoplasmatic as well (Figure 3). Expression of CEACAM 5 was associated with presence of CEACAM 6 expression (P<.001). A correlation between CEACAM 1 and CEACAM 5 expression (P = 0.113) or CEACAM 6 was not observed (P = 0.09).

A correlation with clinico-pathological data revealed no significant association with any parameter for CEACAM 1 except a correlation with distant metastasis (P = 0.008). CEACAM 5 and 6 expression was correlated with a positive lymph node status (P = 0.017 and P = 0.046, respectively) and distant metastasis (P<0.001) (Table 1).

**Table 1:** Immunohistochemical expression of CEACAM 1, 5 and 6, serum expression (ELISA) and correlation with patients' demographic characteristics and clinico-pathological data.

		Tissue Micro Array (IHC)										Serum Expression (ELISA)								
	N	CEACAM 1			CEACAM 5			CEACAM 6			n	CEACAM 1			CEACAM 5			CEACAM 6		
Variables		neg.	pos.	P*	neg.	pos.	P*	neg.	pos.	P		neg.	pos.	pP	neg.	pos.	P*	neg.	Pos.	P
Total	137	51	86		50	87		38	99		46	35	11		31	15		35	11	
		37.2%	62.8%		36.4%	63.5%		27.7%	72.3%			76.1%	23.9%		67.4%	32.6%		76.1%	23.9%	
Sex				.074			.140			0.222				.718			.781			.306
Male	82	26	56		26	56		20	62		21	17	4		17	4		14	7	
		31.7%	68.3%		31.9%	68.1%		24.3%	75.7%			81.0%	23.5%		81.0%	19.0%		66.7%	33.3%	
Female	55	25	30		24	31		18	37		25	18	7		18	7		21	4	
		45.5%	54.5%		43.1%	56.9%		32.1%	67.9%			72.0%	38.9%		72.0%	28.0%		84.0%	16.0%	
Age. Years				.245			.129			.392				.264			.351			.624
≤60	74	30	44		23	51		19	55		15	14	1		10	5		11	4	
		40.5%	59.5%		31.3%	68.7%		25.8%	74.2%			93.3%	7.1%		66.7%	33.3%		73.3%	26.7%	
>60	63	21	42		27	36		19	44		31	21	10		24	7		24	7	
		33.30	66.7%		42.9%	57.1%		29.5%	70.5%			67.7%	47.6%		77.4%	22.6%		77.4%	22.6%	
Tumor stage				.777			.886			.326				.222			.158			.430
T1	7	2	5		2	5		2	5		1	0	1		1	0		1	0	
		28.6%	71.4%		33.3%	66.7%		28.6%	71.4%			0.0%	100.0%		100.0%	0.0%		100.0%	0.0%	
T2	37	12	25		15	22		14	23		6	5	1		6	0		6	0	
		32.4%	67.6%		41.7%	58.3%		37.8%	62.2%			83.3%	20.0%		100.0%	0.0%		100.0%	0.0%	
T3	85	33	52		31	54		21	64		30	21	9		22	8		22	8	
		38.8%	61.2%		36.0%	64.0%		24.4%	75.6%			70.0%	42.9%		73.3%	26.7%		73.3%	26.7%	
T4	8	4	4		3	6		0	7		9	8	1		5	4		6	3	
		50.0%	50.0%		33.3%	66.6%		0.0%	100.0%			88.9%	12.5%		55.6%	44.4%		66.7%	33.3%	
Nodal status				.732			**.017**			**.046**				.230			.846			.863
Negative	55	21	34		26	29		23	32		24	14	10		19	5		19	5	
		38.5%	61.5%		47.8%	52.2%		38.4%	61.6%			58.3%	71.4%		79.2%	20.8%		79.2%	20.8%	
Positive	82	29	53		22	60		21	61		22	20	2		14	8		15	7	
		35.9%	64.1%		26.8%	73.2%		25.5%	74.5%			90.9%	10.0%		63.6%	36.4%		68.2%	31.8%	
Grading				.555			.331			.325				.827			.497			**.016**
G1	7	4	3		4	3		4	4		3	2	1		3	0		3	0	
		57.1%	42.9%		57.1%	42.9%		50.0%	50.0%			66.7%	50.0%		100.0%	0.0%		100.0%	0.0%	
G2	58	21	37		18	40		16	42		28	21	7		20	8		25	3	
		36.2%	63.8%		30.6%	69.4%		26.9%	73.1%			75.0%	33.3%		71.4%	28.6%		89.3%	10.7%	
G3	72	27	45		28	44		18	54		15	10	5		12	3		8	7	
		37.5%	62.5%		38.5%	61.5%		25.4%	74.6%			66.7%	50.0%		80.0%	20.0%		53.3%	46.7%	
M				**.008**			**<.001**			**<.001**				.729			.056			.**002**
negative (N0) (n)	119	39	80		35	84		25	94		36	26	10		28	8		31	5	
%		32.8%	67.2%		24,40%	70.6%		21%	79%			72.2%	38.5%		77.8%	22.2%		86.1%	13.9%	
positive (N1) (n)	18	12	6		15	3		13	5		10	8	2		5	5		3	7	
%		66.7%	33.3%		83,30%	16,70%		72,20%	27,80%			80.0%	25.0%		50.0%	50.0%		30.0%	70.0%	

**Figure 4:**
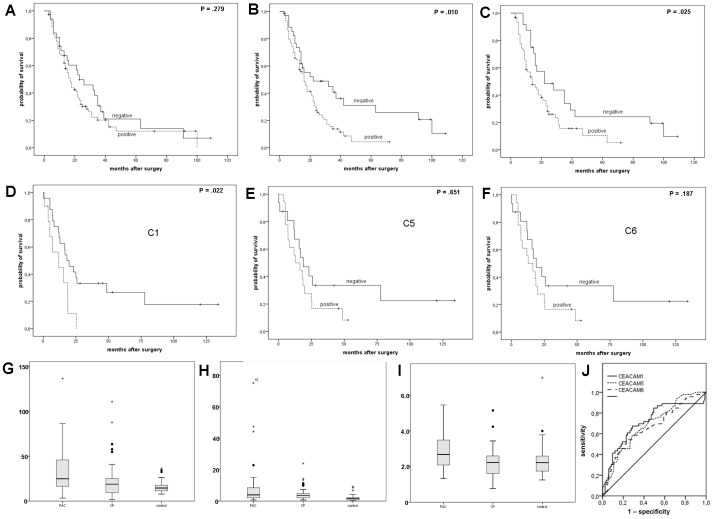
Kaplan-Meier survival analysis (log-rank test) for correlation between overall-survival (OS) and CEACAM expression on the TMA. (A–C). CEACAM 1 positive patients showed a median OS of 17.0 months (14.0–20.1 months), CEACAM 1 negative 23.0 months (9.5–36.5 months, P = .279). OS of CEACAM 5 positive patients was 16.0 months (12.8–19.2 months) vs. 22.0 months (4.1–47.9 months) (p = .025). CEACAM 6 positive patients showed an OS of 14.0 months (7.9–20.0) vs. 22.0 months (5.6–38.4 months, P = .010). Survival curves for correlation between overall-survival and CEACAM serum expression (D–F). CEACAM 1 positive patients showed an OS of 11.8 months (2.4–25.2) vs. 18.3 months (10.0–26.2 months, P = .022). Median OS for patients positive for CEACAM 5 was 15.7 months (2.4–32.3 months) vs. 18.6 months (8.7–28.6 months, P = .651), and median OS for patients with CEACAM 6 positivity was 18.8 months (9.5–28.1 months) vs. 12.8 months (3.3–22.3 months, P = .187). Boxplots for CEACAM 1 serum expression (G), CEACAM 5 (H) and CEACAM 6 (I) compared pancreatic ductal adenocarcinoma (PDAC), chronic pancreatic (CP) and healthy blood donors (BD). Receiver operating curve (ROC) for CEACAM serum expression (J).

The Kaplan-Meier survival analysis showed no correlation between the CEACAM 1 expression and the overall (OS) or disease-free survival (DFS), respectively. Patients with a positive CEACAM 5 and/or 6 expression had a shortened OS and DFS (P = 0.025 and P = 0.007, P = 0.010 and P = 0.030, respectively) (Figure 4A–C, Table 2). In patients with positive expression for all three CEACAM proteins no significant differences in DFS or OS compared with those patients that were negative for all CEACAM subtypes (P = 0.144 and P = 0.742, data not shown) was observed.

**Table 2:** Overall and disease-free survival.

			Overall survival			Disease free survival		
			Negative	positive	p	Negative	positive	p
IHC	CEACAM 1	Median (95%CI)	23.0 (9.5–36.5)	17.0 (14.0–20.1)	.279	9.0 (4.6–13.4)	7.0 (5.6–8.2)	.308
	CEACAM 5	Median (95%CI)	22.0 (4.1–47.9)	16.0 (12.8–19.2)	**.025**	13.0 (11.3–14.7)	6.0 (5.1–6.7)	**.007**
	CEACAM 6	Median (95%CI)	22.0 (5.6–38.4)	14.0 (7.9–20.0)	**.010**	13.0 (6.8–19.2)	7.0 (5.8–8.2)	**.030**
Serum samples (ELISA)	CEACAM 1	Median (95%CI)	18.3 (10.0–26.2)	11.8 (2.4–25.2)	**.022**	12.0 (4.6–18.4)	12.5 (5.0–19.1)	.467
	CEACAM 5	Median (95%CI)	18.6 (8.7–28.6)	15.7 (2.4–32.3)	.651	13.4 (4.5–22.4)	12.0 (3.9–21.4)	.192
	CEACAM 6	Median (95%CI)	12.8 (3.3–22.3)	18.8 (9.5–28.1)	.187	11.9 (5.6–21.0)	13.1 (4.5–20.4)	.091

Kaplan-Meier survival analysis (log-rank test) depending on CEACAM 1, 5 and 6 expression in immunohistochemistry (IHC) and serum samples (ELISA) for patients with pancreatic ductal adenocarcinoma.

### CEACAM 1, 5 and 6 in serum

CEACAM 1 values in PDAC were not elevated compared with patients with CP, but were higher compared to BD (PDAC median 33.0 µg/l, range 3.3–136.7 µg/l; CP median 23.1 µg/l, range 1.8–110.1; BD median 16.1 µg/l, range 7.8–36.5 µg/l; P = 0.059 and P<0.001, respectively). Similar results were found for CEACAM 5: serum values were higher in the PDAC group compared to BD, but not for CP (PDAC median 8.5 µg/l, range 0.7–75.2 ng/ml; CP median 4.8 µg/l, range 0.7–24.0; BD median 1,9 µg/l, range 0.2–9.2 µg/l; P = 0.122 and P = 0.002, respectively). Patients with PDAC showed elevated CEACAM 6 serum expression compared to both, CP and BD (PDAC median 2.90 µg/l, range 1.34–5.46 µg/l; CP median 2.25 µg/l, range 0.77–5.15; BD median 2.34 µg/l, range 1.25–6.99 µg/l; P = 0.06 and P = 0.029, respectively). In none of the performed analyses, a significant difference between CP and BD was detectable (Figure 2G–I).

Receiver operating characteristic curves were used to establish the sensitivity-specificity relationship for CEACAM 1, 5 and 6. The optimal cut-off values were determined by Youdens-Index calculation. The area-under-the-curve (AUC) for CEACAM 1 was 0.711 (cut-off value 184.1 µg/l), for CEACAM 5 0.689 (cut-off value 1.95 µg/l) and for CEACAM 6 0.664 (cut-off value 3.58 µg/l) (Figure 2J) The sensitivity of CEACAM 1 in detecting PDAC was 53.5% with a corresponding specificity of 54.7%. For CEACAM 5, the sensitivity is 79.1% with a specificity of 44.2%. Sensitivity of CEACAM 6 was 47.0% with a specificity of 82.6%. The AUC for a combination for all three CEACAMs showed no improvement compared with the determination of each of the CEACAMs alone (AUC 0.680, data not shown).

For a correlation between CEACAM serum values with clinico-pathological data, the serum values were divided into a low level (<75^th^ percentile) and a high level group (≥75^th^ percentile). High CEACAM 6 values with presence of distant metastasis (P = 0.009) and grading (P = 0.019) (Table 1). A Kaplan-Meier survival analysis calculated with the previous mentioned cut-off values showed a significantly prolonged overall-survival in patients with a low serum expression of CEACAM 1 (P = 0.022) (Figure 2D–F, Table 2). Comparing those patients that showed elevated serum levels for all three CEACAM subtypes to those who showed normal CEACAM serum values revealed no significant differences in DFS and OS (data not shown). Elevated serum levels of CEACAM were not found to be correlated with increased tissue expression in any CEACAM subtype (data not shown).

### Multivariate analysis

In the multivariate cox regression analysis, grading, lymph node status and distant metastasis were found to be independent prognosticators for overall survival in the TMA (Table 3). None of the analyzed CEACAM subtypes reached statistical significance in the multivariate analysis, neither in the IHC nor in the ELISA analysis.

**Table 3:** Multivariate cox-regression analysis for immunohistochemical analysis (IHC) and serum analysis (ELISA).

	IHC				Serum samples (ELISA)			
	significance	HR	95%CI Min	95%CI Max	significance	HR	95%CI Min	95%CI Max
sex	.480	.824	.481	1.410	.654	.752	.216	2.618
age (<65 yrs vs. >65 yrs)	.876	.958	.562	1.633	.591	.731	.233	2.294
pT group (T1/2 vs. T3/4)	.626	1.150	.655	2.020	.753	1.227	.343	4.388
pN	**.016**	2.064	1.146	3.717	.402	.563	.147	2.161
M	**.005**	3.157	1.546	6.705	.401	2.447	.304	19.722
Grading (G1 vs. G2/3)	**.001**	2.410	1.409	4.123	.392	1.659	.521	5.283
CEACAM 1	.423	1.300	.684	2.471	.059	3.971	.950	16.595
CEACAM 5	.086	1.860	.915	3.780	.952	1.040	.296	3.649
CEACAM 6	.071	1.797	.952	3.391	.147	.435	.142	1.339

HR  =  Hazard ratio. T  =  tumor stage. N  =  lymph node stage. M  =  distant metastasis.

## Discussion

The function of CEACAM expression in malignant tumors is still under debate. Several studies that focused either on immunhistochemical or serum expression of one of the numerous CEACAM molecules were previously published [25], [41]–[46]. Here, we assessed the clinical relevance of CEACAM 1, 5 and 6 expression in both, immunohistochemical and serum analysis in patients with PDAC. In addition, we implemented a CEACAM knock down xenograft model for assessment of the potential functional role of CEACAM expression in PDAC.

In our immunohistochemical TMA analysis we found that the majority of tumors expressed CEACAM proteins. About 70% of all analyzed tumor spots showed an expression for either CEACAM 1, 5 or 6, or a combination of all of them. In univariate analysis, CEACAM 5 and 6 expression were correlated with lymph node metastasis. The survival analysis revealed both a shortened overall and disease free survival in patients with a high CEACAM 5 or 6 expression.

Additionally, we quantified CEACAM 1, 5 and 6 in serum of patients with PDAC. Compared to serum samples of healthy blood donors, CEACAM values of all subtypes were elevated. CEACAM 5 and 6 values were higher in PDAC than in patients with CP. Similar to the IHC results, we found that an elevated CEACAM 5 expression correlated with lymph node metastasis and increased CEACAM 6 values were correlated with the presence of distant metastasis and tumor grading. CEACAM 1 was detectable in IHC as well as in blood serum, but a correlation with clinic-pathological data was not evident in our analysis. Interestingly, when serum concentrations were evaluated as predictors for OS, only CEACAM 1 was associated with a shortened OS in the Kaplan-Meier survival analysis.

We were not able to find a correlation between tissue expression and elevated levels in the blood serum of these patients. This might have several reasons: For example, the mere expression of the proteins does not have to result in an increased shedding of them. Furthermore, flushing of the shedded molecule into the blood stream might be a consequence of the disruption of anatomical barriers surrounding host tissues and endothelial cells. Taken together, the mechanisms regulating the shedding of CEACAMs and their dissemination into the surrounding tissue and their entry into the blood system are barely understood and further investigations are needed.

CEACAM 5 is widely used as a serum marker in patients with PDAC in the clinical routine work-up. Our analysis showed a significant correlation between CEACAM 5 and the patients' lymph node status alone but not with survival or any additional clinico-pathological parameter. Therefore, the question arises, whether CEACAM 5 determination preoperatively is of use in the clinical routine work-up. As shown in the serum analysis, the predictive value for primary diagnosis of PDAC with CEACAM determination in the serum alone is unsatisfying, according to our analysis, the sensitivity and specifity is poor.

CEACAMs were shown to be overexpressed in numerous other tumor entities, e.g. colon, breast and lung cancer [17], [20], [21]. CEACAM 1 expression, for example, was found to be associated with a poor clinical outcome in patients with non-small cell lung cancer (NSCLC) and neuroendocrine pancreatic tumors which suggests a pro-tumorgenic effect when the protein is up-regulated [19], [47].

The biological role of CEACAM expression in PDAC is still widely unknown. Since CEACAM expression in normal tissue has pro-angiogenetic effects, regulation of the cell adhesion and may play a part in regulating apoptosis, these effects could also be of importance in tumor progression when CEACAM expression is up-regulated within the tumor cells [8], [9], [48]–[50]. However, increased CEACAM expression is not only seen in malignant, but also observed in inflammatory tissue. One of the physiological roles of the molecule might be pro-inflammatory, which could explain the missing difference between PDAC and CP in our serum analysis [51].

Expression of CEACAM 6 was not only seen in invasive PDAC but also in pancreatic epithelial neoplasia (PanIN) lesions which are considered as tumor precursors, but without an invasive growth [43]. Unfortunately, so far no data exist, whether PanIN lesions with a high CEACAM 6 expression show a higher or faster conversion rate into malignant tumors than PanINs without CEACAM 6 expression. This could rather be of interest whether CEACAM 6 itself promotes a transformation of benign tumor lesions into an aggressive invasive carcinoma. Moreover, an analysis of further CEACAM subtypes would be interesting, since our analysis suggests protumorgenic effects of CEACAM 1 and 5 as well. So far data of CEACAM 1 and 5 expressions in PanIN do not exist.

Even with a prospective database, due to the study design, the obtained data is of retrospective character, which may result in impairment of the significance of those results. With IHC and ELISA studies we are not able to determine, whether CEACAM proteins have a direct effect on local and distant tumor progression or if the revealed statistical correlations are an epiphenomenon of a different process being active in tumor-host interactions. We therefore established a xenograft mouse model with a CEACAM knock down variant of the previously established cell line PACA 5061. Flow cytometry analysis and immunohistochemistry before and after tumor cell injection into the mice showed a stable CEACAM knock down of >90% of all observed CEACAM subtypes. The overall survival in the mice with the CEACAM knock down cell line was significantly prolonged compared to the wild type cell line. This suggests a direct influence of CEACAM-meditated functions in tumor progression. Previous studies already showed anti-tumor effects when CEACAM 6 targeted therapies were used [44], [52]. Binding of Fab antibody fragments against CEACAM 5 and/or CEACAM 6 led to reduced tumor growth in xenograft mouse models and was found to be associated with increased chemoresistance against gemcitabine [41], [53]. In our experiments, we focused on a whole knock down, not only of CEACAM 6. Tumor growth was slower in the knock down cell line and led to an increased overall survival in the knock down group. The interactions between human and murine CEACAM molecules were studied previously [16]. Mice itself do not express CEACAM 5 and 6 subtypes but homo- and heterophilic interactions between different CEACAM subtypes were described extensively [54], [55]. Thus, it is not surprising that CEACAM knock down effects could be observed in the murine xenograft model even without CEACAM 5 and 6 expressions in the mice.

The observed effect of a more aggressive tumor growth in those cells with CEACAM expression is in concordance with the observed effects in the clinical data. As previously shown, tumors with an increased CEACAM expression have to be generally considered as more aggressive and seem to be correlated with a poor prognosis for the individual patient. In most adenocarcinoma (e.g. colon carcinoma), main clinical complication (and ultimately cause of death) is distant metastasis (to lung, liver, bone marrow, etc.) whereas in pancreatic carcinoma locally recurrent tumors and intraperitoneal carcinomatosis is of major importance. Interestingly, the xenograft model used in this study seems to model this situation: The CEACAM knockdown primary tumors showed a slower, less aggressive growth prolonging the animals OS, whereas distant metastasis to the lung increased. These findings are in concordance to our in vitro findings showing the CEACAM knock down cells a higher proliferative and migratory potential than the control cells. Obviously, CEACAMs have no clear protumorigentic or tumorsupressive function in PDAC moreover the mediated effects are of higher complexity. On the one hand, the local tumor growth is significantly impaired in the subcutaneous compartment while at the same time the number of distant metastases increases. Similar effects could be observed for different types of cell surface proteins, like EpCAM whose role of tumor progression is of comparable complexity. Probably, the effect of epithelial-mesenchymal-transition is of some importance in this context. However, we were not able to detect any increased expression in the canonical EMT drivers ZEB1 and ZEB2 in the CEACAM knock down tumors or decreased expression of E-Cadherin or cytokeratin. Obviously, CEACAM expression itself is not correlated with the expression of the canonical EMT drivers as mentioned above. However, as our results suggest, CEACAM expression has a direct influence on the tumor progression and metastatic behavior but without affecting the expression of EMT markers.

So far, the exact functional role of the CEACAM molecules in PDAC is still not fully understood, though we were able to show distinct functional aspects of CEACAM interactions in vitro and in vivo. However, these finding may help understand the inconclusive results that were revealed, not only in our study, with respect to CEACAM expression and the individual patients' prognosis.

## Supporting Information

Figure S1 **Immunohistchemical staining of murine xenograft tumors for markers of epithelial-mesenchymal transtition (EMT).**
(TIF)
