# Supplementary figures and images for: Carcinoembryonic Antigen-Related Cell Adhesion Molecules (CEACAM) 1, 5 and 6 as Biomarkers in Pancreatic Cancer
Source: PLoS One. 2014 Nov 19;9(11):e113023. doi: 10.1371/journal.pone.0113023 (PMC4237406; doi:10.1371/journal.pone.0113023)

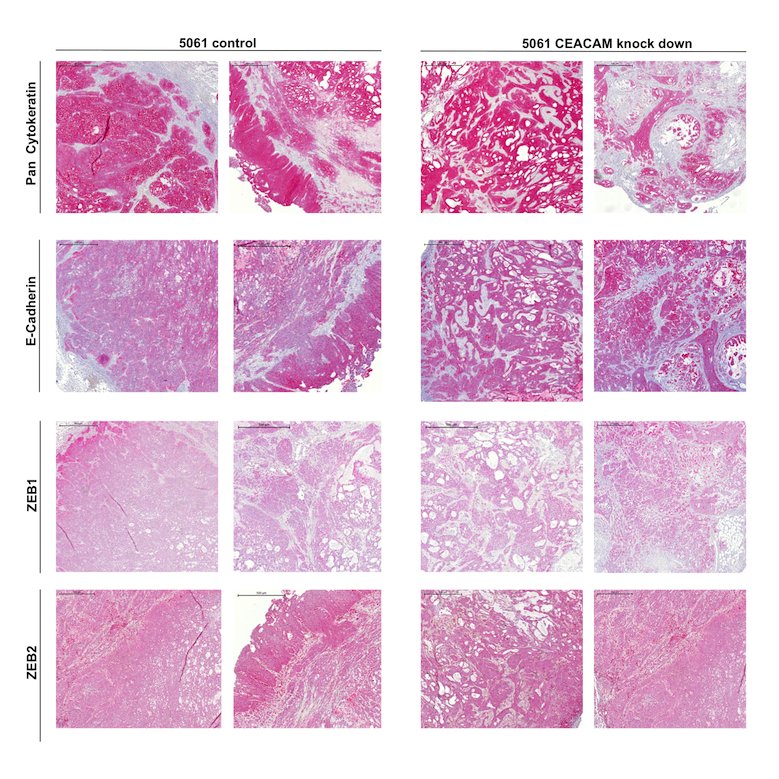

Supplement: Figure S1 — Immunohistchemical staining of murine xenograft tumors for markers of epithelial-mesenchymal transtition (EMT). (TIF) [file pone.0113023.s001.tif]
